# Mikulicz Disease Revealing IgG4-Related Tubulointerstitial Nephritis: A Case Report and Literature Review

**DOI:** 10.3390/reports9020181

**Published:** 2026-06-10

**Authors:** Lissethkaren Alvarez Vargas, Celia Rodríguez Tudero, Elena Jiménez Mayor, Avinash Chandu Nanwani, Esperanza Moral Berrio, Juan Daniel Díaz García, Arturo Villalobos Navarro, Emily Rosario Chamorro Asto, Michael Cieza Terrones, José C. De La Flor

**Affiliations:** 1 Department of Nephrology, Peruvian University Cayetano Heredia and Cayetano Heredia National Hospital, Lima 15102, Perumichael.cieza@upch.pe (M.C.T.); 2 Department of Nephrology, Hospital Universitario de Salamanca, 37007 Salamanca, Spain; crodrigueztudero@usal.es; 3 PhD Program in Surgery, Faculty of Medicine, University of Salamanca, Faculty of Medicine, University of Salamanca, 37007 Salamanca, Spain; 4 Department of Nephrology, Hospital Santa Bárbara, 42005 Soria, Spain; ejimenezmay@saludcastillayleon.es; 5 Department of Nephrology, Hospital General de Fuerteventura, 35600 Canarias, Spain; achanan@gobiernodecanarias.org; 6 Department of Nephrology, Hospital General Universitario de Ciudad Real, 13005 Ciudad Real, Spain; emoral@sescam.jccm.es; 7 Department of Nephrology, Hospital General de México “Dr. Eduardo Liceaga”, Mexico City 06720, Mexico; jdiazg@clinic.cat; 8 Department of Nephrology and Renal Transplantation, Hospital Barros Luco Trudeau, Santiago de Chile 8900091, Chile; 9 Department of Pathology, Instituto Nacional de Salud del Niño, Lima 1399, Peru; emily.chamorro@upch.pe; 10 Department of Nephrology, Hospital Central de la Defensa Gómez Ulla, 28047 Madrid, Spain; 11 Department of Medicine and Medical Specialties, Faculty of Medicine, Alcalá University of Alcalá, 28805 Madrid, Spain; 12 Health Sciences Doctoral Program, Faculty of Medicine, Alcala University, 28805 Madrid, Spain

**Keywords:** Mikulicz disease, tubulointerstitial nephritis, IgG4-related kidney disease

## Abstract

**Background and Clinical Significance:** IgG4-related disease (IgG4-RD) is a chronic fibroinflammatory, immune-mediated multisystem disorder that can mimic neoplastic, infectious, or autoimmune conditions. Among its head-and-neck manifestations, IgG4-related dacryoadenitis and sialadenitis, historically referred to as Mikulicz disease, should be distinguished from the classical *Mikulicz syndrome*, which describes secondary lacrimal and salivary gland enlargement due to other systemic disorders. Renal involvement, most commonly in the form of IgG4-related tubulointerstitial nephritis (IgG4-TIN), is less frequent but carries major prognostic implications because delayed diagnosis may lead to irreversible kidney damage. **Case Presentation:** A 49-year-old man with no relevant past medical history presented with a 2-year history of intermittent polyuria and foamy urine. Laboratory testing revealed advanced kidney dysfunction, with serum creatinine of 4.2 mg/dL, estimated glomerular filtration rate of 16 mL/min/1.73 m^2^, and proteinuria of 2874 mg/day. Physical examination showed bilateral parotid enlargement, upper eyelid edema, lacrimal gland enlargement, and sicca symptoms, raising suspicion for IgG4-related dacryoadenitis and sialadenitis (*Mikulicz disease*). Further work-up demonstrated marked eosinophilia, polyclonal hypergammaglobulinemia, and significantly elevated serum IgG4 levels (3180 mg/dL), while infectious serologies and autoimmune studies were negative. Kidney biopsy revealed plasma cell-rich tubulointerstitial nephritis with lymphoplasmacytic and eosinophilic infiltrates, interstitial fibrosis, tubular atrophy, and more than 40 IgG4-positive plasma cells per high-power field, supporting the diagnosis of IgG4-related tubulointerstitial nephritis in the setting of systemic IgG4-RD. Treatment with prednisone followed by mycophenolate mofetil led to improvement in glandular manifestations and a partial reduction in proteinuria, but renal recovery remained incomplete. The patient subsequently developed a severe pulmonary infection complicated by sepsis and oligoanuric acute kidney injury superimposed on chronic kidney disease, and ultimately progressed to end-stage kidney disease requiring chronic maintenance hemodialysis. **Conclusions:** This case highlights that a *Mikulicz disease* phenotype may represent the initial manifestation of systemic IgG4-RD and should prompt evaluation for extraglandular involvement, particularly renal disease. In patients with glandular enlargement, eosinophilia, hypergammaglobulinemia, and unexplained renal dysfunction, IgG4-RD should be actively considered. Kidney biopsy remains essential for diagnostic confirmation and prognostic assessment, as delayed recognition may result in irreversible renal damage and progression to end-stage kidney disease.

## 1. Introduction and Clinical Significance

IgG4-related disease (IgG4-RD) is a fibroinflammatory, immune-mediated, multisystem disorder characterized by organ enlargement, pseudotumoral lesions, and progressive fibrosis, with the potential to cause irreversible organ damage if not recognized early [1,2]. Its broad clinical presentation frequently masquerades as neoplastic, infectious, or other inflammatory conditions, significantly contributing to the diagnostic delays often encountered in clinical practice [1,2]. Within this spectrum, involvement of the lacrimal and major salivary glands is referred to as IgG4-related dacryoadenitis and sialadenitis, an entity historically known as Mikulicz disease [2,3,4]. This designation should be distinguished from the classical term Mikulicz syndrome, which has historically been used to describe bilateral enlargement of the lacrimal and salivary glands secondary to other systemic disorders, such as Sjögren’s syndrome, sarcoidosis, infections, or lymphoproliferative diseases [2,4]. Therefore, when glandular involvement represents an organ-specific manifestation of IgG4-RD, the most appropriate term is IgG4-related dacryoadenitis and sialadenitis (Mikulicz disease) [2,4]. According to cohort studies, 22% of patients with IgG4-related dacryoadenitis and sialadenitis present with extraglandular systemic involvement. Among salivary gland manifestations, the parotid gland is the most frequently affected site. Orbital involvement is also a recognized manifestation within the spectrum of IgG4-related disease and may occur in association with lacrimal gland enlargement and other ophthalmic findings [5].

From an immunopathological perspective, IgG4-RD is associated with a complex immune response involving B cells and plasmablasts, follicular helper T cells, regulatory T cells, and cytotoxic CD4+ T-cell subpopulations, all of which contribute to both IgG4 class switching and tissue fibrosis [1,2]. Histologically, the diagnosis is based on a characteristic triad consisting of dense lymphoplasmacytic infiltrates, storiform fibrosis, and obliterative phlebitis, together with an increased number and proportion of IgG4-positive plasma cells within the affected tissue [3]. These features are essential for distinguishing IgG4-RD from other autoimmune, inflammatory, or lymphoproliferative disorders that may act as clinical and histological mimickers [2,3].

Renal involvement in IgG4-RD is less common than pancreatic or glandular disease, but it represents a clinically relevant manifestation. According to several international cohort studies, the prevalence of renal involvement ranges from 9% to 12%. Among these manifestations, IgG4-related tubulointerstitial nephritis (IgG4-TIN) is the most common form. Buglioni et al. reported a frequency of 94% in a biopsy-based cohort of 125 patients, although other forms of IgG4-related kidney disease have also been described [6,7,8]. IgG4-TIN usually presents with progressive renal dysfunction, generally non-nephrotic proteinuria, a bland urinary sediment, and, in some cases, hypocomplementemia or suggestive cortical radiological abnormalities [7,8]. Diagnosis requires integration of renal histopathology with clinical, laboratory, or radiological findings consistent with systemic IgG4-related disease [6,7,8].

The diagnosis of IgG4-related tubulointerstitial nephritis is based on the presence of a plasma cell-rich TIN pattern on kidney biopsy together with at least one supportive clinical, laboratory, or imaging feature, as summarized in Table 1.

In this context, we present the case of a patient with IgG4-related dacryoadenitis and sialadenitis (Mikulicz disease) as an extrarenal manifestation associated with biopsy-proven IgG4-related tubulointerstitial nephritis. This case highlights the importance of the differential diagnosis with other systemic diseases, the value of a multidisciplinary evaluation, and the central role of histopathology in diagnostic confirmation, particularly in patients with multiorgan involvement and advanced renal dysfunction [2,3,7,8].

## 2. Case Presentation

A 49-year-old male, with an unremarkable personal and family medical history, presented to the emergency department with advanced renal dysfunction and proteinuria. Review of his clinical history revealed intermittent nonspecific urinary symptoms, including polyuria and foamy urine, for approximately two years before diagnosis. A retrospective review of laboratory records from twelve months prior confirmed preserved kidney function, with a serum creatinine level of 0.86 mg/dL and no evidence of proteinuria or albuminuria. Several months before admission, he developed bilateral parotid enlargement, upper eyelid edema, lacrimal gland enlargement, and sicca symptoms, which prompted evaluation for systemic IgG4-related disease.

Preliminary outpatient laboratory investigations demonstrated advanced renal insufficiency, with a serum creatinine (sCr) level of 4.2 mg/dL and an estimated glomerular filtration rate (eGFR) of 16 mL/min/1.73 m^2^. Urinalysis revealed a bland urinary sediment, without hematuria or leukocyturia, although significant non-nephrotic-range proteinuria was detected (2874 mg/day), prompting hospital admission for further diagnostic evaluation.

Although urinary symptoms had been present intermittently for approximately two years, they remained nonspecific and did not initially prompt nephrological evaluation. In contrast, the subsequent development of bilateral lacrimal and salivary gland enlargement raised suspicion for systemic IgG4-related disease and ultimately led to the diagnostic work-up. The chronological sequence of the patient’s disease course is summarized in Table 2.

**Figure 1:**
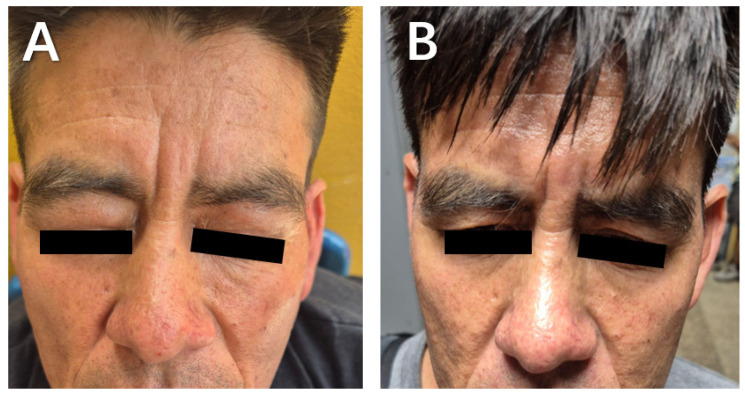
Frontal view of the patient before and after immunosuppressive treatment. (**A**) At admission, bilateral upper eyelid edema is evident, with marked folds and an infiltrative appearance of the supraorbital region. (**B**) After one month of treatment, clinical improvement of the eyelid edema and a reduction in soft tissue volume in the orbital region can be observed.

Physical examination was notable for bilateral parotid gland hypertrophy and prominent upper eyelid edema. Ocular evaluation revealed findings consistent with moderate-to-severe keratoconjunctivitis sicca, characterized by a markedly diminished Schirmer test (right eye: 2 mm; left eye: 4 mm) and a reduced fluorescein tear break-up time (TBUT < 8 s), alongside palpable lacrimal gland enlargement. The remainder of the systemic examination was unremarkable. Given the clinical constellation of bilateral sialadenitis, dacryoadenitis, and sicca symptoms, IgG4-related dacryoadenitis and sialadenitis (historically referred to as Mikulicz disease) was the primary diagnostic consideration, necessitating an expanded work-up to investigate the underlying etiology. These clinical findings were highly suggestive of this entity, as illustrated in Figure 1.

Among the most relevant laboratory findings at admission were markedly elevated urea (124 mg/dL) and serum creatinine (4.57 mg/dL) levels, accompanied by significant eosinophilia (1.46 × 10^3^/µL). Immunological testing revealed a markedly increased serum IgG4 concentration of 3180 mg/dL (reference range, 3–201 mg/dL), together with a low IFN-γ/IL-4 ratio of 0.12, consistent with a predominantly Th2-skewed immune response. Serological testing for human immunodeficiency virus, hepatitis B virus, hepatitis C virus, and syphilis (VDRL) was negative. No hypocomplementemia was detected, and the autoimmune work-up was also negative, including antinuclear antibodies, antineutrophil cytoplasmic antibodies, anti-SSA/Ro, anti-SSB/La, anti-glomerular basement membrane antibodies, anti-PLA2R antibodies, and cryoglobulins. Overall, these findings made common infectious and autoimmune etiologies unlikely, as shown in greater detail in Table 3.

Serum protein electrophoresis revealed hyperproteinemia, with an increased beta-2 fraction of 5.9 g/L (reference range, 2–4 g/L) and marked hypergammaglobulinemia of 30.5 g/L (reference range, 7–17 g/L). Serum immunofixation showed a polyclonal increase in immunoglobulins, arguing against an underlying monoclonal gammopathy. Beta-2 microglobulin was markedly elevated at 24.76 mg/L (reference range, 0.8–2.34 mg/L), and total serum protein was increased to 89.3 g/L, with elevation of the beta-1 (5.4 g/L), beta-2 (5.9 g/L), and gamma (30.5 g/L) fractions. Taken together, these findings supported a state of diffuse immune activation and polyclonal hypergammaglobulinemia, both of which are frequently described in IgG4-related disease.

Renal ultrasonography showed bilaterally enlarged kidneys (140 mm on the right and 130 mm on the left), diffuse cortical hyperechogenicity, preserved parenchymal thickness (21 mm and 25 mm, respectively), and no collecting system dilatation. Renal enlargement is not a normal finding and may be seen in tubulointerstitial nephritis in the presence of substantial interstitial inflammation, supporting the suspicion of bilateral tubulointerstitial disease. Given the combination of significant proteinuria and subacute and severe deterioration in kidney function, a kidney biopsy was performed to characterize the pattern of renal injury and exclude alternative causes of renal involvement.

**Figure 2:**
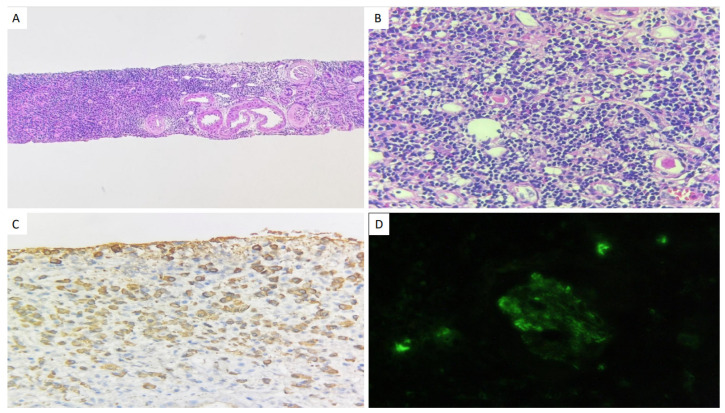
Kidney biopsy findings. (**A**) Hematoxylin and eosin staining, ×50. Extensive tubulointerstitial involvement with moderate fibrosis and severe inflammatory infiltrates. Glomeruli show global sclerosis, and tubules exhibit moderate atrophy. (**B**) Hematoxylin and eosin staining, ×400. Dense interstitial inflammatory infiltrate composed predominantly of lymphocytes, plasma cells, and eosinophils. (**C**) Immunohistochemistry for IgG4, ×400. More than 40 plasma cells with cytoplasmic IgG4 expression are identified per high-power field. (**D**) Immunofluorescence for IgM. All three evaluable glomeruli showed granular deposits in the mesangium and along the capillary loops (+/−).

Light microscopy included seven glomeruli, four of which (57.1%) were globally sclerosed. The interstitium exhibited a severe diffuse inflammatory infiltrate predominantly composed of lymphocytes, plasma cells, and eosinophils, accompanied by interstitial fibrosis involving approximately 30% of the cortical parenchyma and tubular atrophy affecting about 50%, findings consistent with moderate-to-severe chronic tubulointerstitial damage, with focal tubular destruction secondary to inflammation. Immunofluorescence demonstrated granular IgM deposits in the mesangium and capillary loops in three glomeruli, whereas staining for IgG, IgA, C3, and C1q was negative. Immunohistochemical studies revealed more than 40 IgG4-positive plasma cells per high-power field, with additional staining positive in subpopulations for CD3, CD20, and CD138, and a low Ki-67 proliferative index of approximately 5%. On electron microscopy, the interstitium showed dense lymphomononuclear inflammatory infiltration, thickening of the tubular basement membranes with immune-type deposits, epithelial vacuolization of proximal convoluted tubules, tubular atrophy, and luminal obliteration. The glomerular basement membranes were irregular, with associated endothelial swelling, and no organized fibrillary deposits were identified Figure 2.

Taken together, the compatible renal histopathology, the characteristic clinical involvement of the lacrimal and salivary glands, and the markedly elevated serum IgG4 concentration supported the diagnosis of IgG4-related tubulointerstitial nephritis in the context of systemic IgG4-related disease presenting as IgG4-related dacryoadenitis and sialadenitis (Mikulicz disease). The term Mikulicz disease was considered more appropriate than Mikulicz syndrome, as the glandular involvement represented an organ-specific manifestation of IgG4-related disease rather than a secondary manifestation of another systemic disorder.

The patient was started on prednisone at a dose of 1 mg/kg/day. After 1 month of treatment, a clear improvement in the extrarenal manifestations was observed, including a reduction in bilateral upper eyelid edema and orbital soft tissue swelling. Proteinuria also decreased from 3.8 g/day to 2.17 g/day. However, the renal response was incomplete, with the estimated glomerular filtration rate declining from 23.4 to 19 mL/min/1.73 m^2^. In view of the limited renal improvement, mycophenolate mofetil was added at a dose of 500 mg twice daily. At the 3-month follow-up, no significant additional recovery in kidney function was observed, with serum creatinine remaining at 3.4 mg/dL, an estimated glomerular filtration rate of approximately 18 mL/min/1.73 m^2^, and persistent proteinuria of about 2.2 g/day. Over the subsequent 9 months, serum creatinine remained stable at around 3.0 mg/dL, consistent with persistent chronic kidney disease and only partial renal recovery. A dose escalation of mycophenolate mofetil had been planned; however, the patient subsequently developed respiratory deterioration, and imaging studies revealed a lung abscess. His clinical course progressed to sepsis, hemodynamic instability, and worsening kidney function, requiring intensive care unit admission. During this period, he developed oligoanuric acute kidney injury superimposed on chronic kidney disease, with serum creatinine rising to 5.8 mg/dL and urea to 300 mg/dL, which prompted the initiation of continuous venovenous hemodiafiltration. After hemodynamic stabilization, kidney replacement therapy was transitioned to intermittent hemodialysis. He remained hospitalized for more than 2 months, with no recovery of renal function, and ultimately progressed to end-stage kidney disease. Residual urine output gradually declined to approximately 0.5 L/day, precluding reliable quantification of proteinuria. He currently remains on thrice-weekly maintenance hemodialysis without complications.

## 3. Discussion

IgG4-related disease (IgG4-RD) is a chronic, systemic, immune-mediated fibroinflammatory disorder characterized by dense lymphoplasmacytic infiltration rich in IgG4-positive plasma cells, storiform fibrosis, and, in a substantial proportion of cases, elevated serum IgG4 concentrations [1]. Over the past two decades, its recognition as a distinct clinical entity has evolved considerably, and it is now regarded as an important cause of multiorgan pseudotumoral lesions and obliterative fibrosis, predominantly affecting middle-aged adults with a slight male predominance [1]. In this context, our case illustrates an uncommon presentation of IgG4-RD in which bilateral glandular involvement preceded the diagnosis of severe IgG4-related tubulointerstitial nephritis with advanced renal dysfunction. Importantly, although urinary symptoms predated the diagnosis, they were nonspecific and remained clinically unrecognized. The later appearance of the characteristic Mikulicz disease phenotype was the finding that prompted investigation for systemic IgG4-related disease and ultimately revealed the underlying IgG4-related tubulointerstitial nephritis. The relevance of this case extends beyond its initial glandular presentation to include progression to advanced renal involvement confirmed on histology. The combination of bilateral lacrimal and salivary gland enlargement, laboratory findings suggestive of systemic immune activation, and progressive renal dysfunction raised early suspicion of multisystem IgG4-related disease. Accordingly, this case offers an opportunity to address not only the terminological accuracy of the Mikulicz-type glandular phenotype, but also the diagnostic, prognostic, and therapeutic implications of renal involvement in this entity. Within the disease spectrum, involvement of the lacrimal and major salivary glands is currently termed IgG4-related dacryoadenitis and sialadenitis, an entity historically known as Mikulicz disease [4,9]. This terminology should be distinguished from the classical term Mikulicz syndrome, which has historically been used to describe bilateral enlargement of the lacrimal and salivary glands secondary to other systemic disorders, such as Sjögren’s syndrome, sarcoidosis, infections, or lymphoproliferative diseases [9,10]. According to international nomenclature recommendations, when glandular involvement represents an organ-specific manifestation of IgG4-RD itself, the most appropriate designation is IgG4-related dacryoadenitis and sialadenitis (Mikulicz disease) [9]. In our patient, the negativity of anti-SSA/Ro and anti-SSB/La antibodies, together with the absence of histological findings consistent with focal sialadenitis on minor salivary gland biopsy and the lack of evidence for an alternative systemic disorder, allowed Sjögren’s syndrome to be excluded more confidently as the principal diagnosis.

Furthermore, the diagnosis of Mikulicz disease was reinforced by the presence of clinical and serological features highly suggestive of IgG4-related dacryoadenitis and sialadenitis, including persistent bilateral parotid gland enlargement and lacrimal gland hypertrophy of more than 2 years’ duration, a serum IgG4 concentration of 3180 mg/dL—more than 15 times the upper limit of normal—and complete negativity of autoantibodies [4,6]. The revised 2023 diagnostic criteria for IgG4-related dacryoadenitis and sialadenitis have further strengthened this diagnostic framework by broadening the recognized clinical spectrum and incorporating updated histopathological thresholds, thereby providing additional support for classifying the present case within the spectrum of IgG4-RD presenting with a Mikulicz disease phenotype [4].

The serum IgG4 level documented in this patient (3180 mg/dL) deserves particular consideration. Although an elevated serum IgG4 concentration is not diagnostic in isolation, markedly increased levels substantially strengthen the diagnostic likelihood of IgG4-RD and are often associated with a higher inflammatory burden and multiorgan involvement [11,12]. In our patient, the combination of glandular and renal involvement further supports the systemic nature of the disease. In addition, the imbalance between albumin and globulin fractions, eosinophilia, and polyclonal hypergammaglobulinemia with increased beta-2 and gamma fractions on serum protein electrophoresis represent classic laboratory findings that should raise suspicion for this entity [1,12]. The marked elevation in beta-2 microglobulin (24.76 mg/L) is likewise consistent with a state of systemic immune activation, polyclonal B-cell expansion, and advanced renal dysfunction, all of which have been frequently described in active IgG4-RD [12,13].

Renal involvement in IgG4-RD, although less common than pancreatic or glandular disease, is well characterized and occurs in a minority of patients, yet remains of considerable clinical relevance, most commonly in the form of IgG4-related tubulointerstitial nephritis (IgG4-TIN) [7,13]. This entity typically presents with progressive renal dysfunction, a relatively bland urinary sediment, and usually non-nephrotic proteinuria, although other patterns of IgG4-related kidney disease may also be observed [7,13]. In our patient, the histopathological findings on kidney biopsy were decisive for establishing the diagnosis. Light microscopy demonstrated a tubulointerstitial nephritis pattern with a diffuse inflammatory infiltrate rich in lymphocytes, plasma cells, and eosinophils, accompanied by moderate interstitial fibrosis and tubular atrophy. Immunohistochemistry revealed more than 40 IgG4-positive plasma cells per high-power field, far exceeding the accepted histological threshold. In addition, positivity in subpopulations for CD3, CD20, and CD138, together with a low Ki-67 proliferative index, supported the polyclonal inflammatory nature of the infiltrate and helped exclude an underlying malignant lymphoproliferative process [4,7,13].

Although immunofluorescence demonstrated granular IgM deposits in the mesangium and along the capillary loops—a finding that is not characteristic but may occur in cases with concomitant glomerular involvement or overlapping pathological features—these deposits were considered nonspecific because they were not accompanied by significant staining for IgG, IgA, C3, or C1q, nor by histopathological features suggestive of an alternative immune-complex glomerulopathy. Therefore, the overall biopsy findings remained most consistent with IgG4-related tubulointerstitial nephritis [3,8,9]. Taken together, the combination of compatible renal histopathology, characteristic glandular involvement, and markedly elevated serum IgG4 levels established the definitive diagnosis of IgG4-related tubulointerstitial nephritis in the context of systemic IgG4-related disease presenting with a Mikulicz disease phenotype [4,5,9,13].

The finding that 57% of the glomeruli showed global sclerosis at the time of biopsy reflects the extent of irreversible renal damage that had already accumulated before diagnosis. Likewise, the presence of moderate interstitial fibrosis and tubular atrophy suggests that a substantial proportion of the renal injury was no longer reversible at the time treatment was initiated [6,13]. This point is clinically crucial, as the degree of interstitial fibrosis and tubular atrophy on biopsy is one of the main determinants of functional recovery in IgG4-related tubulointerstitial nephritis. In other words, whereas the inflammatory component generally responds to immunosuppressive therapy, established fibrotic lesions are associated with only limited reversibility of glomerular filtration [6,13]. Diagnostic delay is common in IgG4-RD because of its insidious course and its ability to mimic multiple autoimmune, infectious, and neoplastic conditions. This reinforces the need to include IgG4-RD in the differential diagnosis of any otherwise unexplained interstitial nephropathy, particularly when accompanied by glandular involvement, eosinophilia, or hypergammaglobulinemia [1,12,14].

Glucocorticoids remain the cornerstone of induction therapy in IgG4-RD with renal involvement, with prednisone at a dose of 0.6–1 mg/kg/day being the most commonly used regimen in clinical practice [1,12,15,16]. In our patient, prednisone at 1 mg/kg/day induced a partial clinical response, with improvement in eyelid edema and a reduction in proteinuria, but without substantial recovery of kidney function, suggesting an anti-inflammatory effect superimposed on already established chronic structural damage [15,16]. This pattern is consistent with published clinicopathological series, in which many patients show improvement with immunosuppressive therapy, yet renal recovery remains incomplete when significant interstitial fibrosis is already present [7,13]. The addition of mycophenolate mofetil as a glucocorticoid-sparing strategy or as adjunctive therapy in cases of incomplete response has been described in clinical practice, although the available evidence derives mainly from retrospective studies and accumulated experience in IgG4-RD cohorts, with no robust disease-specific trials in IgG4-related tubulointerstitial nephritis [1,12]. In our patient, therapeutic escalation was limited by the development of a lung abscess, which progressed to sepsis and hemodynamic instability, ultimately requiring intensive care unit admission, invasive mechanical ventilation, and the initiation of kidney replacement therapy. Severe infections are recognized complications of immunosuppressive treatment in this disease, particularly in patients with impaired renal function and significant comorbidity [12]. Although rituximab was not used in this case, its efficacy in inducing remission in refractory or relapsing disease is well documented, with the additional potential advantage of reducing cumulative glucocorticoid exposure [17]. The fact that this patient ultimately progressed to chronic maintenance hemodialysis underscores the impact of delayed diagnosis, pre-existing tubulointerstitial fibrosis, and the superimposed septic episode on the final renal outcome.

This case underscores the importance of considering IgG4-related disease in the differential diagnosis of any patient with a Mikulicz disease phenotype and otherwise unexplained renal dysfunction. Measurement of serum IgG4 levels and, above all, kidney biopsy remain essential tools for diagnostic confirmation. Early recognition may allow the timely initiation of specific therapy and improve prognosis, particularly before advanced interstitial fibrosis, tubular atrophy, and irreversible loss of renal function become established [1,13,14].

## 4. Conclusions

This case highlights that bilateral lacrimal and salivary gland enlargement consistent with Mikulicz disease may represent the initial manifestation of systemic IgG4-related disease rather than an isolated glandular disorder. When accompanied by renal dysfunction, eosinophilia, and polyclonal hypergammaglobulinemia, prompt evaluation for extraglandular involvement is warranted, particularly for IgG4-related tubulointerstitial nephritis, given its prognostic significance. Kidney biopsy remains essential for diagnostic confirmation and assessment of chronic irreversible damage, emphasizing that delayed diagnosis may result in incomplete renal recovery and progression to end-stage kidney disease.

## Figures and Tables

**Table 1:** Diagnostic criteria supporting the diagnosis of IgG4-related tubulointerstitial nephritis (IgG4-TIN).

Category	Description
Primary criterion	Presence of plasma cell-rich tubulointerstitial nephritis (TIN) pattern on kidney biopsy.
Clinical criterion	Clinical evidence of IgG4-related disease in at least one other organ.
Laboratory criterion	Elevated serum IgG or IgG4 levels, or polyclonal hypergammaglobulinemia.
Imaging criterion	Characteristic renal imaging findings, including small peripheral cortical nodules, round or wedge-shaped low-density lesions, or diffuse patchy renal involvement on computed tomography or magnetic resonance imaging.

**Table 2:** Clinical timeline of the patient’s disease course.

Time	Clinical Events
Two years before diagnosis	Nonspecific urinary symptoms, including polyuria and foamy urine.
Twelve months before diagnosis	Documented normal kidney function (serum creatinine 0.86 mg/dL) with no proteinuria or albuminuria.
Several months before admission	Development of glandular enlargement (ogressive bilateral parotid and, lacrimal gland) and sicca symptoms.
Admission	Severe renal dysfunction and heavy proteinuria, associated with eosinophilia, polyclonal hypergammaglobulinemia, and markedly elevated serum IgG4 levels.
Kidney biopsy	Histopathological confirmation of IgG4-related tubulointerstitial nephritis (IgG4-TIN).
Treatment response	Improvement of glandular manifestations and partial reduction in proteinuria, with limited renal recovery despite immunosuppressive therapy.
Later complication and outcome	Lung abscess complicated by sepsis and acute kidney injury, ultimately leading to end-stage kidney disease requiring maintenance hemodialysis.

**Table 3:** Laboratory data at admission.

Parameter	Value	Reference Range
Hemoglobin/Hematocrit	15.4 g/dL/47%	13–17 g/dL/40–50%
MCV/MCH	92 fL/30.4 pg	80–96 fL/27–32 pg
Leukocytes	9.7 × 10^3^/µL	5–10 × 10^3^/uL
Neutrophils	5.61× 10^3^/µL	2–7.5 × 10^3^/uL
Eosinophils	1.46 ×10^3^/uL	0.0–0.5 × 10^3^/uL
Lymphocytes	1.73× 10^3^/µL	1.5–3.5 × 10^3^/uL
Platelets	285 × 10^3^/µL	150–400 × 10^3^/uL
Glucose	83 mg/dL	75–110 mg/dL
Urea	124 mg/dL	19–43 mg/dL
Creatinine	4.57 mg/dL	0.6–1.3 mg/dL
Albuminuria 24 h	376 mg/24 h	<30 mg/24 h
Proteinuria 24 h	3800 mg/24 h	<150 mg/24 h
Albumin/Globulin ratio	0.7	>1
Albumin	3.36 g/dL	3.5–5.0 g/dL
Globulins	4.74 g/dL	2.0–3.5 g/dL
Serum IgG4	3180 mg/dL	3–201 mg/dL
ANA/ANCA/Anti-Ro/Anti-La	Negative	
C3/C4	96 mg/dL/21 mg/dL	79–152 mg/dL/14–38 mg/dL
Serology: HIV, HBV, HCV, VDRL	Negative	

Abbreviations: MCV, mean corpuscular volume; MCH, mean corpuscular hemoglobin; ANA, antinuclear antibodies; ANCA, antineutrophil cytoplasmic antibodies; HIV, human immunodeficiency virus; HBV, hepatitis B virus; HCV, hepatitis C virus; VDRL, Venereal Disease Research Laboratory.

## Data Availability

No new data were created or analyzed in this study. The data used to support the findings of this study are available from the corresponding author on request (contact J.C.D.L.F., jflomer@mde.es).
